# Leveraging a Province-Wide Electronic Health Record System to Integrate Health Promotion into Routine Hospital-based Care in Alberta, Canada- A Research Protocol for Evaluation

**DOI:** 10.5334/ijic.9697

**Published:** 2026-08-07

**Authors:** Kamala Adhikari, Gary F. Teare

**Affiliations:** 1 Cancer Prevention and Screening Innovation, Primary Care Alberta (formerly: Provincial Population and Public Health, Alberta Health Services), CA; 2 Department of Community Health Sciences, Cumming School of Medicine, University of Calgary, CA

**Keywords:** integrate health promotion, screening, brief intervention and referral, healthcare settings, modifiable risk factors, electronic health records

## Abstract

**Objective:**

The Integrating Prevention into Connect Care for Health (IPiC-Health) initiative embeds a Screening, Brief Intervention, and Referral (SBIR) approach for health promotion into routine inpatient and ambulatory care, facilitated by the province-wide, EPIC™-based, electronic clinical information system in Alberta. The SBIR approach supports patients in achieving behaviour change regarding tobacco and alcohol use and physical inactivity. This methodological article describes the research plan to evaluate the implementation and effectiveness of SBIR intervention.

**Methods:**

This effectiveness-implementation hybrid study includes a quasi-experimental design for quantitative assessment of SBIR implementation (adoption/coverage) and effectiveness (behaviour change and health service use) outcomes. Each participating site recruits control patients for 6 months, followed by 15 months intervention. Patients are followed up for 12 months after recruitment to assess effectiveness outcomes. Mixed methods are used to assess implementation uptake, process, and experience.

**Expected results and implications:**

Recruitment and data collection activities are ongoing in participating sites. This effectiveness-implementation research will strengthen evidence and implementation guidance to inform spread and scale up of the approach across Alberta’s hospitals and beyond. An electronic record of patient’s risk factors can improve patients’ care and population health by making these data readily available to support care decisions.

## Background

Making use of opportunities for health promotion and disease prevention when people seek healthcare is critical to increasing the impact of healthcare on population health. Only 10–20% of what makes people healthy is attributable to the treatment of illness, while 30% has to do with individuals’ health behaviours [1]. According to recent reports of the World Health Organization, 22.3% of the world’s population use tobacco, 27.5% of the adult population do not meet the recommended level of physical activity, and 42.6% of the population are current consumers of alcohol [2,3,4]. In Alberta, 18% of the population smoke cigarettes, 28% engage in greater than low risk levels of alcohol drinking, and 70% do not get enough physical activity, as defined by the Canadian guidelines for a healthy lifestyle [5]. Tobacco consumption, alcohol misuse, and physical inactivity are leading modifiable risk factors for preventable chronic diseases (e.g., cancer, cardiovascular disease, respiratory diseases) and poor outcomes (e.g., complications and increased healthcare use or costs) [6,7,8,9,10,11,12,13,14,15]. According to the Canadian Population Attributable Riskfor Cancer study, in 2015, 28% of new cancer cases diagnosed in Canada were attributable to tobacco use, 5% to drinking alcohol, and 9% to physical inactivity [5]. Together, the three risk factors account for approximately 20% of total healthcare expenditures [15]. Importantly, these risk factors are modifiable by raising awareness and encouraging behaviour change; these negative impacts could be prevented or reduced by supporting patients’ health behaviour change [15,16,17]. Of the estimated 40% of cancers that are preventable, the greatest proportion are attributable to modifiable risk factors, including tobacco and alcohol use and insufficient physical activity [14].

Although incorporating screening for behavioural health risk factors and promoting healthy behaviour change are important goals for primary care, there are also compelling reasons to incorporate health promoting interventions within hospital-based care. Hospital settings provide opportunities to support a large proportion of patients to modify their risk to prevent chronic diseases and improve treatment outcomes. Tobacco and alcohol use and physical inactivity are common (43%–90%) among patients attending hospitals [18,19]. Also, these risk factors are common among diverse subpopulations (e.g., some minority populations and socioeconomically disadvantaged people). These subpopulations, who often lack access to family physicians and primary care, are over-represented in the hospital settings. Healthcare providers are a trusted source of information about health risks, and patients who are currently experiencing ill health are more responsive to health advice for lifestyle behaviour change [20]. Significantly, a large proportion of total healthcare costs are driven by the costs of hospital care related to diseases associated with these factors [7]. Although general medicine guidelines recommend counselling on these risk factors as standard practice, it is not reliably integrated into hospital care routines.

The health promoting hospitals and health services (HPH) concept, developed by the World Health Organization over 30 years ago based on the principles of the WHO Ottawa Charter for Health Promotion, aims to reorient health services from a solely curative focus toward more holistic health care that includes health promotion [21]. In Alberta, Canada, our Cancer Prevention and Screening Innovation group has implemented several initiatives during the last 10 years to integrate screening, brief intervention, and referral (SBIR), pertaining to modifiable risk factors for cancer, as an approach for health promotion in hospital settings [22,23]. Our earlier health promotion pilots evaluated the implementation feasibility of SBIR using a paper-based guidance and documentation approach [22] However, these pilot initiatives as well as the existing global literature have lacked rigorous measurement of the health and healthcare impacts of this kind of HPH initiative as well as evidence-based guidance to support spread and scale out of implementation [24,25]. Research evidence on the effectiveness of HPH is limited, particularly in Canada, with no high quality peer reviewed studies yet published in English [24]. Most existing studies relied on surveys, interviews, and case studies to explore the enablers and barriers of HPHS projects. However, they offered limited evaluation of intervention implementation processes, impacts, mechanisms of impact, and contextual factors, and often lacked the use of appropriate study designs [24]. To enable large-scale integration of health promotion activities into routine hospital care, clinical leaders and decision makers need both good evidence of their effectiveness as well as evidence-informed implementation guidance pertinent to regular clinical settings. There is a clear need for more rigorous evaluation with focus on effectiveness and implementation outcomes and dissemination of results to inform the integration, spread, and sustainability of prevention-oriented hospital care.

The traditional research approach that focuses on testing an intervention under ideal conditions (i.e., efficacy and effectiveness trials) followed by real-world effectiveness trials before considering translation into regular clinical practice takes a significant amount of time and resources. The field of implementation science has offered a potential means to accelerate uptake of innovations, through the methodologies for effectiveness-implementation hybrid studies [26,27]. Hybrid studies can simultaneously incorporate formative (process and developmental) and summative evaluation methods, providing a comprehensive understanding of the real-world effectiveness of an intervention, the implementation process or strategies used to deliver it, and implementation outcomes [26,27]. Hybrid studies can offer more useful information for decision-makers, balance internal and external validities, and speed the translation of research findings into routine practice.

By early 2025, Alberta completed a multi-year province-wide roll out of a single EPIC™ electronic clinical information system (locally called “Connect Care”) in all of its hospitals, ambulatory care clinics, public health services, continuing care facilities, and laboratory services. This technology offers a sustainable opportunity to integrate, SBIR approach to health promotion within routine patient care workflows, guided by and documented in an electronic information system. The electronic capture of data related to routine care also makes evaluation of the implementation process and the downstream impacts on patient’s health and healthcare system far more feasible.

Our current initiative in Alberta, Canada – the Integrating Prevention into Connect Care for Health (IPiC-Health) project has two-fold objectives: (a) to integrate SBIR, to identify and address tobacco use, alcohol use, and insufficient physical activity, within Connect Care-facilitated patient care workflows in a variety of acute and ambulatory care settings, as a routine component of care; and (b) to demonstrate the feasibility and impacts, on patient outcomes and health system costs, of SBIR implementation within these settings. The IPiC-Health initiative intends to strengthen the quality and generalizability of effectiveness evidence and implementation guidance to inform spread and scale out of the approach to a wide variety of clinical programs. This methodological paper describes the research plan to rigorously evaluate the implementation and effectiveness outcomes of SBIR intervention.

## Overview of the Program

### Description of intervention and settings

**Figure 1:**
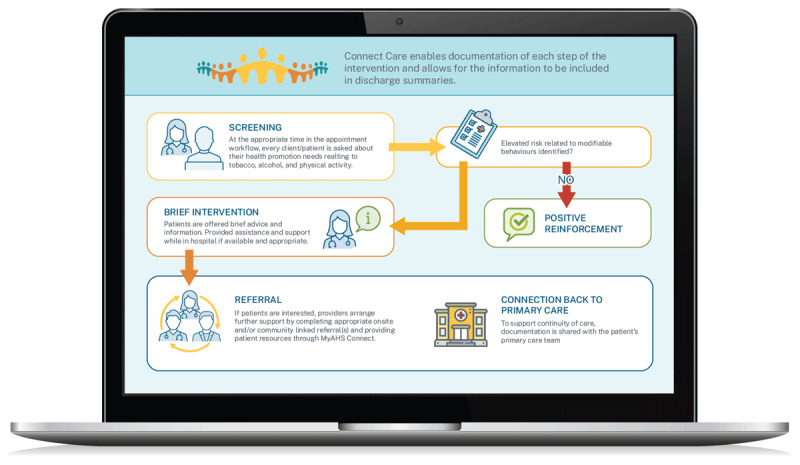
SBIR workflow.

The IPiC-Health initiative is a three year project (January 2023-December 2025) that engages a variety of ambulatory and inpatient healthcare sites and providers in delivering SBIR for health promotion as a routine component of patient care. These sites provide care to patients with a variety of acute and chronic health issues, including rheumatological conditions, surgery, diabetes, pneumonia, asthma, cardiac diseases, trauma, and geriatrics. This initiative leverages the province-wide electronic (“Connect Care”) clinical information system to help integrate the SBIR actions and documentation sustainably into routine care. Screening involves using standardized screening questions to identify patients who use commercial tobacco, consume alcohol at levels that increase risk, or who participate in insufficient physical activity [28,29,30,31,32,33]. The brief intervention is a conversation that providers have with patients who have elevated risk on one or more of those modifiable risk factors. The brief conversation aims to educate patients about the health risks, identify patient readiness to consider a change in behaviour, and to encourage them to take action. Providers are then able to offer educational resources (e.g., a brochure containing QR codes/links to relevant websites) that provide behaviour change information and support. Providers can also refer patients to support services available in their community (e.g., Alberta Quits program for tobacco cessation) (Figure 1).

Connect Care contains evidence-informed, Alberta-relevant screening tools, risk algorithms, patient education materials, and referral information pertinent to tobacco use, alcohol misuse, and physical inactivity. We are working to optimize the use of these tools/resources by collaborating with the clinical teams, patients, and Connect Care experts to learn and demonstrate how Connect Care can facilitate practical delivery of SBIR for these risk factors within various clinical programs and workflows. We work with each clinical team to ensure that providers can confidently conduct brief educational/motivational conversations with patients using patient education materials and conversation guides/scripts developed/embedded within Connect Care. We identify health behaviour change support services available to patients in Alberta and within the local areas of the participating hospitals and ensure that information about them is accessible through Connect Care to provide patient information and referrals.

### Anticipated implementation challenges and strategies employed

Based on lessons learned from our previous SBIR pilots and our review of the global literature, we anticipated several factors that can hinder or help the implementation of SBIR in hospitals. These include: change fatigue and competing priorities, patients’ perceived or experienced stigma and providers’ discomfort to discuss behavioural risk factors with patients, functionality of the Connect Care system to support this work, limited capacity/motivation to provide preventive care in hospital settings, resources/support available to deliver SBIR or Connect Care support, and the adaptability of SBIR within a variety of clinical workflows [22,23,34,35]. Accordingly, several strategies (tools, training, technical assistance, and quality improvement methods) are planned and implemented together with stakeholders at each participating site to address the anticipated barriers and facilitate successful SBIR implementation and adaption [35,36]. The tools include a SBIR implementation guide, a single page information sheet (“cheat sheet”) to guide providers in delivering and documenting SBIR. Training of the clinical team members involves 1:1 or group in-person education and information sessions on how SBIR contributes to patient recovery and health (tailored to the specific patient populations/conditions served by each site), and how to complete and document SBIR using Connect Care. Technical assistance includes continuous engagement, by the research and implementation support team, with clinical team members and their leaders to build trust and to co-design the tailored support they need. Research staff are embedded in clinical sites to undertake the research activities of our project and to support clinical staff by offering regular coaching and troubleshooting concerning the delivery and documenting of SBIR. Quality improvement support involves continuous assessment of implementation, identifying “what worked’ or didn’t, and why, and provision of feedback reports on the clinical teams’ progress in implementing SBIR. We work proactively with clinical teams to refine the implementation strategies. The implementation sites and units were intentionally selected to ensure that no other major initiatives were occurring concurrently

Clinical teams were given flexibility to tailor the implementation process and the timing of brief intervention conversations to best align with patient needs and existing clinical workflows. In acute care settings, for example, these conversations were typically initiated during the later stages of hospitalization, such as in the days leading up to discharge, during discharge preparation, or once patients had passed the most acute phase of their stay. When clinical teams determined that the hospital setting was not appropriate for delivering the intervention to particular patients, providers were empowered to use their clinical judgment to assess the suitability of the intervention. In situations where hospitalized patients were not ready or able to engage with substantial information, patients were given the option to decline the intervention or to receive it during subsequent clinical encounters.

## Methods

### The design and key features of implementation and evaluation

**Figure 2:**
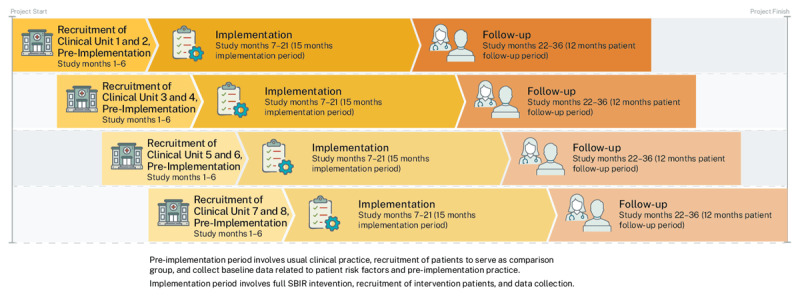
Overview of Intervention Implementation Design.

We employ implementation-effectiveness hybrid study using mixed-methods [26]. This includes quasi-experimental design for quantitative assessment of SBIR implementation (adoption/coverage of intervention) and effectiveness (patient behaviour change and health service use) outcomes. We use both quantitative and qualitative methods to evaluate the implementation processes and providers’ experience of integrating SBIR into their workflows and patients’ experience of the SBIR intervention and of health behaviour change. Each participating site participates in a 6-month pre-implementation period, followed by 15-month SBIR implementation research period. Patients are followed up at 1, 6, and 12 months after they receive the SBIR intervention (Figure 2). Clinical sites that had used Connect Care for at least 6 months prior to recruitment into this project, and where the team identified health promotion and disease prevention as a priority, and expressed interest in participating in the study were engaged to participate.

During the 6-month pre-implementation period, our onsite research coordinators recruit a no-intervention (control/comparison) group of study participants and conduct risk factor screening on recruited patients using paper-based version of the same screening tools as those in Connect Care that will be used during SBIR implementation. These patients do not receive risk factor screening by clinical team members and are not offered brief intervention and referrals. The 15-month SBIR implementation serves as the intervention period of the study during which patients are recruited to participate in the intervention group. Note that all patients served by the participating clinics/units during the intervention period are *eligible* to receive the SBIR intervention, regardless of whether or not they agree to participate in the research study. Over the course of the 15 months intervention period, as the SBIR practice is increasingly embedded into routine care, we expect a growing proportion of the patients served by the participating clinical teams will receive SBIR. Enrolled research participants are followed up over the course of 12 months (1, only for intervention group, 6, and 12 months) for collection of behaviour change and health services utilization data. During the monitoring of participant recruitment, any control participants who had subsequent visits to intervention sites and received the intervention at that time were excluded from the study.

### Research plans for evaluating implementation outcomes and process

*Study design and participants:* We use a quasi-experimental interrupted time-series design, covering a 6-month pre-implementation period and the last 12 months of the 15-month SBIR implementation period. The first three months of the implementation period are not used in analysis because we expect the quality of the SBIR intervention will mature over the first few months during which the clinical team learn to apply the practice.

*Definition and measurement of implementation outcomes*: Intervention reach (number) and patient population coverage (proportion) will be measured for each component of the SBIR intervention. Thus, the number of patients i) screened; ii) advised; and iii) referred in relation to each risk factor will be extracted each month from the Connect Care data. The denominator for the coverage (proportion) measures will be based on the total number of patients seen by a participating unit in the same month.

*Analysis:* For each outcome, we will calculate monthly summaries, aggregated across participating units. A Poisson regression model will be used to estimate the difference between the post-intervention observed rate and the “spontaneous” rate that would have occurred had SBIR been not implemented. The analysis will account for seasonality by decomposing the data in trend, seasonal, and random components, and then removing the seasonal component.

*Implementation process evaluation:* We employ qualitative and quantitative methods using surveys, focus group discussions, and in-depth interviews to understand the SBIR implementation process and patients’ and providers’ experience. This involves assessments of: factors affecting providers’ implementation/adherence to SBIR; providers’ perceptions of SBIR acceptability, appropriateness, and feasibility; providers’ perceptions of their role in patient-centered, preventive care; patients’ experience (acceptability, perceived stigma, and challenges with SBIR and behaviour change). We are using the Consolidated Framework for Implementation Research (CFIR) and Theoretical Domains Framework (TDF) to guide the ongoing assessment of implementation and obtain a comprehensive understanding of barriers/facilitators, behavior/practice change of healthcare providers, and the contextual factors that influence SBIR implementation [37,38,39]. A detailed data collection plan for both implementation and effectiveness outcomes are shown in data collection matrix table (Table 1).

**Table 1:** Data Matrix.


OUTCOME CATEGORY	OUTCOMES	MEASURES	DATA SOURCES	DATA COLLECTION METHODS

**Pre-implementation Phase (6 Months Control Period)**				

Baseline, for comparison of SBIR (Screening Brief Intervention, and Referral) effectiveness	Tobacco consumption, alcohol intake, and physical activity levels	Proportion of patients with tobacco consumption, alcohol misuse, and insufficient physical activity levels	Primary data collection from comparison patient group	Paper-based self-administered questionnaire

Pre-implementation (baseline) delivery of SBIR elements by clinical team members.	Providers’ baseline delivery of SBIR elements to patients	Proportion of patients being screened, advised, and referred for each risk factor	Connect Care documentation (electronic health records)	Connect Care data extraction, monthly time points for six months

Baseline	SBIR implementation readiness	Leadership engagement/support, resources for SBIR, providers’ motivation	Primary data collection from providers and organizational leaders	Self-administered questionnaire, interview, and observation

**Implementation Phase (15 Months Intervention Period)**				

Implementation	Providers’ delivery of SBIR to patients: Reach or penetration of SBIR to patients; providers, intervention adherence	Proportion of patients being screened, advised, and referred for each risk factor	Connect Care documentation (electronic health records)	Connect Care data extraction, monthly time points for 15 months

Implementation	Acceptability, appropriateness, and feasibility of SBIR implementation	Proportion of providers being agreed or strongly agreed that SBIR implementation is acceptable, appropriate, and feasible in clinical settings	Primary data collection from providers who implemented SBIR	Survey using a validated tool at six and 12 months of implementation initiated

Implementation	Adherence or adaptation to SBIR implementation and real-world practice change challenges/barriers	Whether providers achieve the goal of screening all patients for the three risk factors and offering brief advice and referral to all eligible patients and if not, why; whether any adaptations were made in implementation, what they were, and why adaptions were made.	Primary data collection from providers who implemented SBIR	Regular conversations/interactions during regular meetings between clinical and research teams using a standardized template.

Implementation	Factors influencing implementation	Unit- and organization-related barriers/facilitators or contextual factors to SBIR implementation	Primary data collection from clinical and administrative leadership (e.g., nurses, unit managers, implementation facilitators)	In-depth interviews at three and nine months after implementation initiated

Patients follow ups: Patients’ experience and perspective	Patient’s knowledge and experience (intervention patient groups)	knowledge about the impacts of the risk factors on health risks and the available services/programs for behavior change; acceptability, perceived stigma, and any challenges with SBIR process or related materials; barriers/facilitators to risk factor-related behavior change.	Primary data collection from patients	Follow up in-depth telephone interviews with a small sample at one months of index hospitalization discharge/visit (recruitment or intervention receive time).

Patient follow ups: Effectiveness of SBIR intervention	Patients’ behavior change status for both control and intervention patient groups	Intention to change behavior, awareness about the problem, commitment to action, uptake to referral programs, active modification of behavior (such as quit attempt, reduce/quit tobacco use, reduce/stop alcohol intake, and initiate/increase physical activity). Covariates for effectiveness outcomes: Sociodemographic/socioeconomic (such as age, sex, marital status, ethnicity, education, employment, income, rurality, immigration-related factors) and overall physical and mental health status.	Primary data collection from patients.	Follow-up telephone interview at six months of index hospitalization discharge/visit. Data collection tool will include the questionnaires guided by the Prochaska model of change process and the risk factor screening questions included in the Connect Care

Healthcare resource use	Hospital readmissions and emergency visits for both control and intervention patient groups	Hospital readmissions and emergency visits during the year after index hospitalization discharge /visit. Reasons/diagnosis for readmissions and emergency visits, and family physician/primary care visits within 1-year of discharge/visit and diagnoses/comorbidities at the time of recruitment to 1-year of index hospitalization discharge/visit.	Administrative health databases: Discharge Abstract Database, National Ambulatory Care Reporting System, and physician claim databases	Data from multiple databases will be accessed through Data and Analytics. and will be linked with the follow-up interviews using the personal health numbers.

**Post-implementation (after Intervention Period)**				

Data collection will continue up to one month post-implementation for patient’s knowledge and experience outcomes, up to six months post-implementation for patient’s behavior change outcomes, and up to one-year post-implementation for healthcare resource use outcomes.				


## Research plans for evaluation of intervention effectiveness outcomes

*Study design and participants:* We use a quasi-experimental study design (pre-post with a comparison group). The intervention group are patients from participating units during the SBIR implementation period who screen at increased risk on any of the three risk factors and received brief intervention and referral support. The comparison group are patients from the same units, during a 6-month pre-implementation period, identified as having increased risk on any of the three risk factors, but who do not receive the SBIR intervention.

Study inclusion criteria for the target patient population to be enrolled in the effectiveness study include: Adult patients (>18 years) who are residents of Alberta, have the ability to engage in the SBIR process and data collection, and are attending the participating sites during the study period. Research assistants enroll the eligible patients in the study after they provide informed consent. The patients with severe disease conditions (i.e., unconsciousness and end-stage care) or those with mental or cognitive incapacity are excluded.

*Outcome definition and measurement for both groups:* Key outcomes are patient behaviour change in relation to the modifiable risk factors and health care utilizations in the year after recruitment to the study. Measurement of patient’s behaviour changes involves: questions on patients’ intention to change behaviour, their uptake of referral services (for those who received referrals), and changes in risk level as measured using the same risk factor screening tools used during the SBIR intervention. We also ask patients about tobacco use reduction, tobacco quit attempts, seven- and 30-day tobacco abstinence. The data are being collected via telephone interviews at 6 months follow-up after the index clinic visit, or discharge from a hospital stay, during which the patient was recruited into the study. A sample of participating patients are also called at 1 month after their hospital/clinic visit to ask them about their experience of the SBIR intervention.

Health care utilization measures include hospital readmissions and emergency visits within one year of the index discharge/visits. These outcomes are of particular importance to facilitate outcome comparisons with other studies as well as because they are important to provide cost-effectiveness assessments to support intervention spread/scale decisions by health system administrators. The health care utilization data will be obtained from linked administrative health databases accessed through the Department of Data and Analytics, the custodian of these routinely collected healthcare data (Table 1).

Data on potential confounding variables including sociodemographic and socioeconomic status for both groups are collected during the 6 months patient follow-up interviews. Diagnosis and comorbidity related variables will be obtained from the administrative health databases. A detailed data collection plan is shown in data collection matrix (Table 1).

*Sample size:* SBIR intervention can increase smoking quit rate at 6-months by 11% [40], reduce alcohol misuse rate at 5-months by 19% [41], and increase patients’ average daily physical activity level by 22% [42]. Based on sample size estimation in relation to tobacco use (with effect size of 11%), we will need a maximum of 1,000 patients in each group (a total sample of 2,000 for controlled and intervention groups) to detect the smallest difference, among all outcomes, between the groups (with a study power of 80% and alpha of 0.05). This is the maximum number needed since tobacco is the least prevalent condition and it will also have the smallest proportion of change in behaviour after intervention, among the three risk factors. We will recruit a total sample of 4,000 (2,000 sample for controlled group and 2,000 for intervention group) to account for the anticipated 50% loss to follow up or nonresponse rate. The expected dropout rate is determined based on our previous paper-based SBIR pilot study [22].

*Analysis:* We will use log-binomial regression models to estimate risk difference and relative risk (for health behaviour change outcomes), and Cox proportional competing risk regression models to estimate hazard rate difference and hazard ratio (for health care use outcomes), to compare outcomes between the two groups adjusting for confounders, such as reasons or diagnosis for visits/admissions, comorbidity, and socioeconomic status.

Cost-effectiveness analysis will document the economic value of the SBIR intervention in order to communicate that value to decision-makers. Cost reduction/avoidance within one year, attributable to SBIR, will be estimated based on the reduction in 1-year readmissions and emergency visits, compared to the non-intervention comparison group. Estimates of this economic impact will be provided by applying hospitalization and emergency visit cost data, provided by Canadian Institute of Health Information (CIHI), to the utilization estimates from the above-mentioned models. Intervention implementation cost data are being collected throughout the project.

*Ethical considerations:* This study has received ethics approval from the Health Research Ethics Board of Alberta – Community Health Committee- at the University of Calgary (HREBA.CHC-22-0058).

*Involvement of people with lived experience:* IPiC-Health project has 7 patient advisors, who closely work with the research and innovation team of IPiC-Health project. The patient advisors provide insights based on lived experience to inform SBIR pathways, the research and implementation plan, development of resources, brief conversation scripts, and provide advice on acceptability and appropriateness on all aspects of the research and intervention. The Patients advisors consist of individuals connected to the Alberta Strategy for Patient Oriented Research SUPPORT Unit (AbSPORU) and Patient and Community Engagement Research (PaCER) | University of Calgary-trained patient researchers who have lived experience of care in Alberta’s patient care settings and/or with the risk factors targeted by the SBIR intervention.

## Discussion

### Advantage

The strengths of our method center around the evaluation of both effectiveness and implementation outcomes in a single study to generate effectiveness and implementation evidence that is critical for integrating care in routine practice. Specifically, this study measures the effectiveness of patient behavior changes in response to the SBIR intervention, while simultaneously evaluating the feasibility and acceptability of implementation through process-oriented, mixed methods, guided by implementation science frameworks. We engage with multiple clinical stakeholders (patients, providers, and clinical managers or leaders) at an early stage and throughout the implementation, which allows us to understand potential barriers/facilitators and informs the selection of appropriate implementation strategies for real-world implementation and sustaining the intervention. In addition to the quantitative assessment of implementation feasibility, acceptability, and appropriateness, process-oriented qualitative assessment during the implementation allows to seek feedback from patients and providers or site managers or leaders on implementation potential in their setting and adaptation needs for a better fit in their context, and understand how and why interventions may vary in effectiveness across settings [27,43,44,45]. This approach focuses on identifying why strategies work (or not) and informing the implementation needs or co-plan strategies going forward to support the adoption, adaptation and sustainability of the intervention, as opposed to just demonstrating their impact on reach, adoption, and fidelity [27,43,44,45,46]. The concurrent gathering of both effectiveness and implementation data is critical, otherwise, we may be unaware of crucial contextual factors related to the success or failure of the interventions.

Most of the previous studies of SBIR focused on alcohol use in primary care settings and mainly assessed effectiveness of SBIR in reducing alcohol consumption [25,47,48,49]. However, very few studies have assessed effectiveness outcomes through an equity lens (e.g., age, socioeconomic status, race) and performed ongoing assessments of barriers and facilitators alongside iterative refinement of implementation processes [25]. In addition, the majority of these studies were randomized controlled trials (which, while rigorous, often provide limited guidance on real-world implementation). Other studies have relied on weaker study designs (such as those without control groups), and frequently lacked comprehensive measurement, and the use of theoretical frameworks for evaluation [25]. Our effectiveness-implementation hybrid evaluation approach addresses the existing gaps by employing a pragmatic quasi-experimental study design (with a control group), integrating mixed methods and implementation science frameworks to systematically assess both outcomes/effectiveness and implementation processes. This approach increases the feasibility of conducting the study in a real-world setting while maintaining the rigour and generalizability of the evidence and provide evidence-informed implementation guidance to help integrate this intervention as routine patient care practice in real-world healthcare settings. This dual focus on effectiveness and implementation outcomes is an evidence-based approach that has been used in different studies conducted in various healthcare settings [26,27,43,50]. To our understanding, the use of this approach is new in the context of evaluating EHR-facilitated SBIR intervention in healthcare settings to address the modifiable risk factors for cancer and chronic diseases of patients.

The judicious integration of evaluation of effectiveness and implementation outcomes in a single study can help practically integrate research in healthcare settings and address healthcare needs. This provides more useful information for decision-makers, increases the generalizability of the findings, and improve the speed of moving research findings into routine adoption, compared to pursuing these lines of research independently [26,27,43,50]. However, keeping effectiveness and implementation research separate and sequential overlooks complexity, limits the generalizability and slows down the translation of knowledge into routine practice [26,27,43]. Effectiveness research alone is more affected by generalizability because of a failure to document and attend to contextual factors influencing effectiveness.

### Disadvantage

Given the different, multiple priorities and methods involved in evaluating effectiveness and implementation outcomes, the IPiC-Health study has potential challenges that require special attention, which are similar to the existing literature that used hybrid study designs [26,27,36,44,45,46,47,51,52,53]. The focus on evaluating both outcomes, using mixed methods, can lead to competing priorities and resource allocation challenges, and the potential of de-prioritization of either of the outcomes or associated activities [27,43,46,51]. Given the complexity of and multifaceted nature of the study design and implementation process, clinical buy-in or engagement, defining and measuring implementation outcomes, and identifying and reporting implementing strategies can be challenging [27,46,52]. While local adaptation can improve contextual fit and uptake, it can produce differences in fidelity within local adaptations; thereby leading to variations in the effectiveness of the intervention. Collection and organization of various types of quantitative and qualitative data from multiple, different sources at multiple times within a complex clinical implementation context requires ongoing attention from multiple members or experts [51]. To mitigate these challenges, the IPiC-Health study involves a specialized, interdisciplinary project team, including expertise in implementation science, methodology, ethical considerations, data integration, clinical contexts, and project management [36]. The project team continuously collaborates with the participating settings to ensure a smoother implementation process, uses electronic medical records and health administrative data, and documents the ongoing interaction between project and clinical teams to reduce the burden of data collection [35,52,53,54]. Additionally, project staff are embedded in research sites to perform the research participant recruitment and primary data collection, and provide implementation supports [35,36]. The project team carefully plans, executes, and monitors the study activities regularly to adequately address the needs of both aspects of research and balance internal and external validities.

Self-administered questionnaires are used to assess risk factors in the control group. These same questionnaires are integrated into the Connect Care system to support provider-led screening within routine clinical workflows, which are used for risk factor screening in the intervention group. Social desirability bias can be present when patients respond to questions about their health-related behaviours. However, we have no reason to expect the bias in responses to the risk factor questions will be different between the control and the intervention groups. Additionally, the follow ups at 6 months and 12 months to measure behavior change and healthcare use outcomes, respectively, may underestimate the intervention’s ultimate effects, as the outcomes may take a longer period to manifest. However, existing literature has demonstrated measurable changes within these timeframes [40,41,42,55], and demonstrating near-term impact was a key criterion for the grant funding mechanism supporting this project.

We acknowledge that effective prevention care ideally addresses the full prevention continuum; however, this was beyond the scope of the current project, which focused on establishing improved processes for delivering preventionrelated care within hospital settings. Within our intervention approach, patients who expressed interest in behavior change were encouraged and supported to access relevant communitybased services and supports. Because direct referral pathways within the Alberta health system were available only for certain behavior change needs (specifically smoking cessation and highrisk alcohol consumption), some referrals were necessarily informational and patientdirected. Hospital-anchored prevention pathways are simply one of many on-ramps that need to be made available to patients/people to support the promotion of health and the reduction of illness and its related health system costs. Ultimately, health promotion through primary care and through intersectoral partnerships to create supportive health environments in community will be vital to improving population health and reducing avoidable hospitalization.

## Expected Results and Applicability

Thus far 4 inpatient units and 4 ambulatory clinics in Calgary and Edmonton cities in Alberta have participated in this project. Among those, 1 inpatient unit and 4 ambulatory clinics have completed the 6 months control period and started the implementation of SBIR, and remaining units/clinics are in the 6 months control phase. We have recruited a total of 2015 patients as control research participants (i.e., medium or high-risk level on any of the three risk factors). Of those, 1000 have completed 6 months follow-up calls. A total of 447 patients have received SBIR support from the participating units/clinics, with 175 patients being intervention research participants (i.e., consented for follow up survey). Among those intervention research participants, 48 have completed 1 month follow up survey calls, 75 have completed 6 months follow up calls, and 23 have participated in in-depth qualitative interviews. Approximately 50% of patients are responding to follow-up efforts.

The research participants recruitment and data collection activities are undergoing and key learnings to date are as follows.

- High screening uptake: Providers are consistently screening and documenting for the risk factors.
- Low brief intervention rates: Offering brief advice or engaging patients in conversations about behavior change remains relatively low.
- Low documentation of brief intervention: When brief interventions are provided, they are often not documented in the Connect Care system.
- Referral gaps: Referral pathways—particularly for physical activity and alcohol use—are unclear, and referral functionalities for these risk factors are not currently integrated into the Connect Care system.
- Facilitators of implementation: Implementation is supported by clinical champions, embedded project staff at implementation sites, and ongoing rapid assessment and feedback.
- Participant eligibility challenges: Many patients are ineligible for the intervention or study participation due to critical health conditions particularly in some high acute care settings. This indicates that careful consideration of the setting and patient acute is important when deciding whether to implement SBIR.
- Improved recruitment: Using both in-person and phone-call recruitment strategies has led to an increase in participant numbers.

We will analyze the implementation and effectiveness data once the complete data become available. Findings will be shared with health programs and decision makers, in local national and international conferences, and will be published in the peer review journals. The research findings will strengthen the quality and generalizability of evidence and guide the future implementation, spread, and scale up of this initiative on integration of health promotion and disease prevention-oriented routine care in acute and ambulatory care settings across Alberta’s hospital system. An electronic record of patient’s modifiable risk factors on EHR improves patient’s care and population health by making data available instantly and securely to authorized users (e.g., healthcare professional, patients, researchers, program designers) to make decisions about holistic patient care. Furthermore, this initiative will guide in integrating the broad social determinants of health-oriented care, such as addressing financial and food insecurities, to patients in healthcare settings (i.e., scale-out of the initiative). Overall, rigorous implementation and evaluation methods used in this study enhance the integration of health promotion and disease prevention in healthcare settings, ultimately benefiting patient care and public health.
